# Reports on the Hospitals of the United Kingdom

**Published:** 1910-04-30

**Authors:** Henry Burdett


### REPORTS
### ON THE
### Hospitals of the United Kingdom.

By SIR HENRY BURDETT, K.C.B., K.C.V.O.

### SOME SMALLER HOSPITALS.

### XLI.—HOSPITAL OF ST. CROSS, RUGBY.

This hospital was founded in 1884 by a Mr. Woods, who had it built by his own architect according to his own ideas, after visiting a number of hospitals. Although funds were abundant, the result is an excessive covering of ground with bricks and mortar, with a small but good ward planted here and there at uncertain and irregular intervals, and no hygienic or other advantages to compensate for the great administrative drawbacks. It is a striking example of the wastefulness of providing money for hospital buildings without first taking the precaution to secure and follow the results of experience with the aid of an architect who has been trained adequately in this special department of construction. Much of the hospital is good, and endeavours are evidently made to maintain it in the best of order throughout.

The plan followed has resulted in a great waste of space, and the cost of maintaining the establishment and keeping it clean and smart must be excessive. For the patients, if the management is as good as it is economical, this hospital should be a comfortable and sure refuge to all in need of hospital care. The fittings and the beds and bedding are good, but the patients' lockers are not of a good pattern, and there might be a little more furniture in the wards.

In view of what has been said already, it may not surprise the expert to learn that the accommodation for the nurses is situated in the roof. Much of it is mixed up with that for the maids, and, with the exception of three rooms it is as bad as bad can be. Two nurses occupy the same room, and that room is far too small and without a fireplace. No sufficient bath accommodation is provided, and such as there is is not conveniently situated. It is discreditable to find defective provision for the nurses in buildings of such magnificent proportions, seeing that they have to bear the brunt of the work, and that it is a committee's first duty to take care of the workers in the institution for the efficiency of which they are responsible.

### XLII.—SHEPTON MALLET DISTRICT HOSPITAL.

Established in 1869, this hospital was subsequently rebuilt, but unfortunately the work was not entrusted to a hospital architect or an experienced adviser, and it is faulty in several important respects. The children's ward has no lavatory, bath, or stair accommodation. The staircase is so badly planned as to make it impossible readily to carry a patient upstairs, where most of the wards are situated, and some of the balustrades have to be removed to enable this to be attempted, at discomfort and risk to the patient and the staff. The whole staircase ought to be reconstructed at the expense of the architect. The mortuary is just as little of a mortuary as possible. All the usual appliances and an adequate water-supply are absent. It should be put right and properly equipped without delay.

Everything that devotion and knowledge can secure in the way of efficiency has been done by the matron, Miss Saunders. Every ward, and indeed every department of the hospital, is kept in the most perfect order, and there was not a cupboard or any part of the institution which we did not find to be a pattern of what such places should be, although our inspection was made without notice and came as a great surprise to the authorities. Miss Saunders is an excellent book-keeper, and she takes charge of the operation register and the patients' admission book. She has also presented daily a written report of everything material which takes place in the hospital, which is a model of what these things should be. The book in which these reports are written is a new departure in cottage hospital management, and might be adopted with advantage by every cottage hospital committee throughout the country.

Like many modern hospitals, large and small, this institution has felt the effects of the working of the Workmen's Compensation Act. The authorities, finding that accident cases were coming into the hospital in increasing numbers, and that a workman sometimes gets more money by being unemployed and ill than when well and working, have altered the rules. They now make each accident case of the kind pay for admission to the hospital, a practice which all hospitals should follow without exception. The workpeople, to their honour, gladly agree to pay.

This hospital has a lady, Miss Annie Hyatt, M.B., B.S. London, attached to its staff. Her influence has been good, her skill is gratefully recognised, and she has proved in all repects a source of strength to the institution. Miss Saunders, the matron, has had great and varied experience, having been a Queen's nurse in Manchester, matron to the Bourton-on-the-Water Cottage Hospital, and also a worker in Axminster and elsewhere. She is a good type of what a cottage hospital matron should be, and has excellent method and organising powers.

**The Chorlton Union Infirmary, West Didsbury (Manchester),** will be reported on next week.

